# The Current State of Knowledge on Osteoporosis in Holocaust Survivors and Their Descendants

**DOI:** 10.5041/RMMJ.10523

**Published:** 2024-04-28

**Authors:** Malvina Hoxha, Visar Malaj

**Affiliations:** 1 Department for Chemical-Toxicological and Pharmacological Evaluation of Drugs, Faculty of Pharmacy, Catholic University Our Lady of Good Counsel, Tirana, Albania; 2 University of Tirana, Department of Economics, Tirana, Albania

**Keywords:** Bone health, Holocaust survivors, Jewish survivors, osteoporosis, starvation

## Abstract

**Objective:**

Starvation in early life can cause poor bone health and metabolic aberrations in bone minerals, leading to abnormal bone development. Holocaust survivors have been exposed to starvation and malnutrition before and during World War II. This paper aims to provide the current state of knowledge on the osteoporosis risk in Holocaust survivors and their descendants.

**Methods:**

The PubMed and Scopus databases were searched. Papers that reported original data on the risk of osteoporosis in Holocaust survivors and in their offspring were included in the study.

**Results:**

Ten studies were included in this review. The majority of studies were case-control ones (*n*=7) versus two self-reported and one longitudinal study. Despite the limited cohort numbers and the small number of studies in the literature, the data showed a potential increased risk of osteoporosis in Holocaust survivors and especially in their descendants.

**Conclusions:**

The review of these studies showed a higher prevalence of osteoporosis among Holocaust survivors and their offspring. Knowledge of the trans-generational inheritance of osteoporosis in the descendants of Holocaust survivors should increase the awareness of primary care health workers on osteoporosis screening and early diagnosis and implementation of preventive measures, including adequate vitamin D and calcium supplementation, and pharmacological treatment.

## INTRODUCTION

The Holocaust (Hebrew *Sho’ah*) was the systematic persecution and murder of six million European Jews by the Nazi German regime and its collaborators from 1933 to 1945. Nearly two out of three Jews living in countries under the control or influence of Germany during World War II were executed. A small proportion of survivors were former prisoners of concentration camps, ghettos, and killing centers. In addition, hundreds of thousands of Jews survived the Holocaust by escaping Nazi-occupied territories.

Most Holocaust survivors, regardless the circumstances, lived in constant fear of death and were exposed to significant nutritional deprivation. The type of nutritional deprivation would have differed depending on whether survivors had lived in a work camp, ghetto, extermination camp, had been in hiding, or a combination thereof. Each type of deprivation presented its own dangers. For example, some survivors were fed only with corn or grass pea, the latter of which, if consumed in high quantities, caused severe motor neuron disease and lathyric osteopathy.^1^,^2^

Starvation in infanthood can lead to demineralization of the bones in both sexes. Cooper et al. showed that low vitamin D and calcium levels and nutritional deprivation are predictors of osteopenia in adults.^3^ In addition, different studies have shown the role of environmental factors on bone mass and density in the prenatal, childhood, or adolescent periods.^4^,^5^ A reduced peak bone mass can cause osteopenia and osteoporosis.^6^ Insufficient protein intake in undernourished children can lead to reduced bone mass and delayed skeletal growth.^7^

The term “hunger disease” refers to the effect of starvation, studied by 28 Jewish physicians in the Jewish population of the Warsaw ghetto.^8^ Their research defined the “hunger osteopathy” diagnosed in Warsaw ghetto survivors as a bone disease that flourished during starvation. Autopsies of individuals who died of starvation in the ghetto revealed bone porosity and decalcification.^9^ Although their research was translated from Polish and published in French shortly after World War II, an English translation became generally available only in 1979.

The term “famine disease” was used by Danish researchers in reference to individuals exposed to starvation in concentration camps. A 1970 Danish study listed a number of symptoms of famine disease such as weight loss, hunger diarrhea, hunger edema, and hunger polyuria.^10^ In addition, infection was considered to be a complication or expression of famine disease. Bradycardia, hypotension, depression, insomnia, irritability, excessive fatigue, urolithiasis, muscle pain, hernias, bone decalcification, and back trouble were some of the symptoms of famine disease.^10^ Nutritional deprivation in pregnant women, including calcium and vitamin D, can also lead to premature osteoporosis in adults, premature cartilage ossification, metabolic syndrome, reduced peak bone mass, and more.^11^,^12^ The transformation of mesenchymal cells in osteoblasts and osteoclasts, which leads to successful skeletal development, requires a sufficient supply of vitamins and minerals, nutrients found in milk, meat, and other proteins. However, these basic requirements were not met, and the prolonged nutritional deprivation of the Jewish population in concentration camps and ghettos, especially in pregnant women, led to abnormal fetal skeletal development, osteomalacia, and osteoporosis.^13^–^15^ The risk of falling and having a hip fracture and the prevalence of osteoporosis has been shown to be increased among men and women who lived under the Nazi regime, or in a Nazi-occupied country.^16^

Mental health conditions such as post-traumatic stress disorder (PTSD) and the increased incidence of cancer are the most recognized health issues in Holocaust survivors, whereas osteoporosis risk has not been sufficiently studied. The aim of this study is not only to assess the current state of knowledge on the osteoporosis risk in Holocaust survivors and their descendants, but also to inform on the burden on healthcare systems when this group of patients, especially the second generation of Holocaust survivors, are not currently being proactively identified and treated.

## METHODS

This systematic review was performed according to the preferred reporting guidelines for systematic reviews (PRISMA).^17^

### Study Design

This systematic review sought to report on all known findings in the literature on the osteoporosis risk in Holocaust survivors and their descendants.

### Eligibility Criteria

Our search was made in September 2023 and was not restricted by the year of publication. Studies carried out in any country were included in the review if they contained original data on the risk of osteoporosis in Holocaust survivors, or in their offspring. We excluded reviews, systematic reviews, conference papers, posters, protocols, and letters to the editors. Only papers in English were included.

### Literature Search and Selection of Articles

A search in the PubMed and Scopus databases was performed using the following search terms: “osteoporosis in Holocaust survivors”; “osteoporosis in Jewish survivors”; “osteoporosis in Holocaust offspring”; “concentration camp Jewish survivors and osteoporosis”; “Holocaust ghetto survivors and osteoporosis”; “prevalence of osteoporosis in Holocaust survivors”; and “osteoporosis in Holocaust descendants.” Only studies that fulfilled the eligibility criteria on the risk of osteoporosis in Holocaust survivors and their descendants were included.

### Data Synthesis and Extraction

Ten out of 29 identified papers were included in this systematic review. Duplicate papers were removed, and discrepancies were double-checked and discussed between M.H. and V.M.

## RESULTS

### Overview of Selected Studies

**Figure 1:**
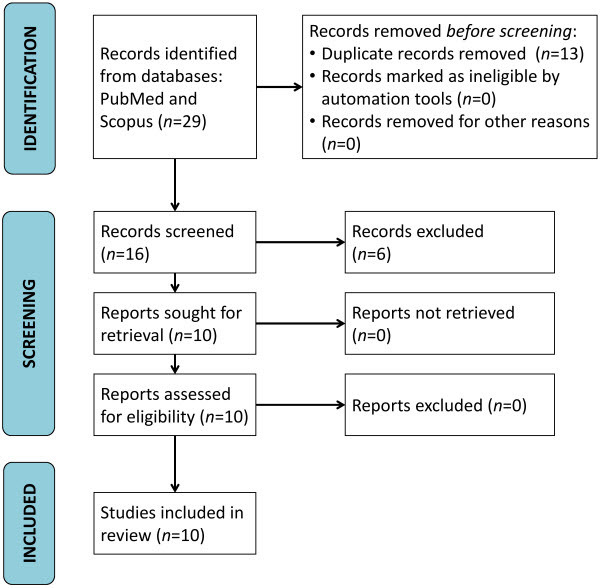
Prisma Flow Diagram of Literature Search and Selection for Articles Included in this Systematic Review.

We identified 29 studies, of which 13 were removed as duplicates. Other studies not fulfilling the eligibility criteria were removed (*n*=6). Only 10 studies met the inclusion criteria (Figure 1). An overview of the data extracted from each study is provided in Table 1.

**Table 1:** Overview of All Studies Included in this Systematic Review.

Ref	Study Type	Population	Bone Parameter Assessed	Outcomes
Thygesen et al. (1970)^10^	Longitudinal study	Four groups: **Group IA:**710 police force members deported from Copenhagen in 1944; serving in Copenhagen in 1948 **Group IB:** 572 MRM living in Copenhagen May-September 1947 **Group II:** 321 MRM with social incapacity studied during 1950–66 **Group III:** 52 MRM without pension and living in Copenhagen or suburbs during January–April 1964	Muscle pain, back pain	**Group IB:** 47% had headache and/or muscular pain **Group II:** 40% had muscle pain mainly in the back and loins, usually at night, related to “restless legs” Two members of group II experienced back “trouble”
Brodsky (2000)^16^	Self-report study	European-born Jewish women ≥60 years old (median age 74)	Unknown	Increased prevalence of self-reported osteoporosis in Holocaust survivors vs controls (33% vs 16%)
Foldes et al. (2003)^18^	Case-control study	Men and women incarcerated in concentration or labor camps	Unknown	Increased prevalence of fractured hips in patients of both sexes who lived in Nazi regime or Nazi-occupied countries
Werner (2003)^19^	Self-report study	530 Holocaust survivors of Nazi ghettos, camps, or in hiding (1933–1945)	History of fracture, joint pain, and back pain	Increased hip fracture prevalence in Holocaust survivors
Marcus and Menczel (2007)^20^	Observational case-control study	73 female Holocaust survivors ≥60 years incarcerated in concentration camps, ghettos, labor camps, children’s institutions, or in hiding	BMD measured in lumbar spine (L2-L4) and both femoral necks	Significantly increased osteoporosis risk in Holocaust survivors
Weisz and Albury (2013)^21^	Observational case study	11 Holocaust survivors living in Australia (5 females and 6 males); all exposed to starvation in early life	GARVAN; FRAX; DEXA	Severe starvation in early life increases osteoporosis risk from gestation and throughout life in males and females
Weisz and Albury (2014)^22^	Case-control study	3 siblings, survivors of the Budapest ghetto	GARVAN; FRAX; BMD; DEXA	Nutritional deprivation during second trimester of pregnancy associated with osteopenia/osteoporosis
Weisz and Albury (2016)^2^	Case study	1 Holocaust survivor and her son	FRAX; DEXA	Data showed generational transfer of metabolic insult and onset of osteoporosis
Mizrahi et al. (2017)^23^	Retrospective cohort study	58 Holocaust survivors vs 82 non-Holocaust survivors hospitalized for consecutive hip fractures aged ≥64 years	Femoral head joint fracture during the inpatient rehabilitation period; FIM	Holocaust survivors demonstrated lower total motor FIM and gain scores at discharge, which could have led to the hip fracture and internal fixation
Weisz (2019)^15^	Observational case series	Holocaust survivors (*n*=4) (diagnosed <70 years old), all exposed to starvation and/or malnutrition Descendants: second generation (*n*=17); third generation (*n*=5)	DEXA T- and Z-scores; GARVAN; FRAX	Potential osteoporosis risk in Holocaust survivors and in second- and third-generation survivors

BMD, bone mineral density; DEXA, dual-energy X-ray absorptiometry; FIM, Functional Independence Measure™; FRAX, fracture risk assessment tool; GARVAN, Garvan Fracture Risk™; MRM, members of the resistance movement; Ref, reference.

Most of the reviewed papers (*n*=7, 70%) were case-control studies that reported bone densitometry (i.e. dual-energy X-ray absorptiometry [DEXZ]) T- and/or Z-scores; Garvan Fracture Risk (GARVAN); the Fracture Risk Assessment Tool (FRAX^®^); or Functional Independence Measure™ (FIM) in Holocaust survivors or their descendants versus controls.^2^,^15^,^18^,^20^–^23^ Two papers (20%) were self-report studies, and only one paper (10%) was a longitudinal study.^10^,^16^,^19^ The increased prevalence of hip fractures among Holocaust survivors was reported in three studies.^18^,^23^,^29^ The time range of publication was 1970 to 2019.

The earliest published study (1970) was performed in Denmark and included former concentration camp prisoners (classified in four groups).^10^ The authors reported that “painful joint” was an irrelevant symptom.^10^ However, the results showed that 40% of the group II cohort (past members of the resistance movement [MRM] with a social incapacity during 1950–66) had “restless legs” and muscle pains mainly in the back and loins.^10^ Two members of group II also had back trouble. Furthermore, 47% of group IB (past MRM and residents of Copenhagen during May–September 1947) suffered headaches and/or muscular pain.^10^ Even though little was known about osteoporosis in the early 1970s, the muscle pain and back troubles reported in this study could have been indicators of the disease.

A 2007 study was conducted in 73 female Holocaust survivors aged 60 years or older who had been in a concentration camp (*n*=36), in a ghetto (*n*=28), in a labor camp (*n*=14), in hiding (*n*=24), or in a children’s institution (*n*=6) in different countries. Data acquired from bone mineral density (BMD) exams of the lumbar spine (L2–L4) and both femoral necks showed an increased risk of osteoporosis among Holocaust survivors.^20^

An observational case series study was carried out in 4 Holocaust survivors, 17 children of survivors, and 5 grandchildren of survivors who had been exposed to starvation and/or malnutrition. The DEXA T- and Z-scores were calculated on the 4 survivors and their descendants, along with GARVAN and FRAX scores.^15^ The author showed a potential risk of osteoporosis in both the second- and third-generation Holocaust survivors.^15^ Earlier, Weisz and Albury reported the generational transfer of metabolic changes in a Jewish woman residing in Australia but born in a Hungarian ghetto while her mother was experiencing severe starvation during pregnancy.^2^ She was diagnosed with osteoporosis in her 50s, and her son had undergone DEXA measurement in his 40s.^2^ In line with these findings, another study reported that of three siblings from one family, one was exposed to nutritional deprivation during the third trimester *in utero*, when the family was incarcerated in a Hungarian ghetto; this sibling developed osteoporosis or osteopenia in later life.^22^

Another observational case study by Weisz and Albury provided data from 11 Holocaust survivors living in Australia (5 females and 6 males) who had been exposed to starvation in early life in concentration camps and ghettos in different countries.^21^ The fracture risk, including GARVAN and FRAX, was reported for all patients. Despite the limited cohort, the data indicated that osteoporosis risk in both sexes increased depending on the type of nutritional deprivation: gestation to infancy, childhood, or young adulthood.^21^ It should be noted that Australia has the highest number of Holocaust survivors per capita outside Israel; hence further studies should be performed on a similar cohort that includes more Jews who immigrated to Australia.^24^,^25^

In a retrospective cohort study performed in Israel in 58 Holocaust survivors aged 64 years or older, the FIM was used to study rehabilitation risk after hip fracture in Holocaust survivors.^23^ For the first time, it was shown that Holocaust survivors had lower total motor FIM and gain scores at discharge, which increased the risk of hip fracture and internal fixation. Another Israeli study in female Jewish survivors aged 60 years or older confirmed a higher prevalence of self-reported osteoporosis and hip fractures in Holocaust survivors versus controls (33% versus 16%).^16^ Foldes et al. and Werner supported these findings and also demonstrated an increased prevalence of osteoporosis and hip fracture in Holocaust survivors of both sexes, who had lived under the Nazi regime, or in a Nazi-occupied country.^18^,^19^

## DISCUSSION

The BMD measured in female Holocaust survivors showed that the risk of osteoporosis was statistically increased in patients who were <17 years in 1945.^20^ A significant portion of peak bone mass (40%) is gained during adolescence.^26^ Hence, undernutrition in early life increases the risk of osteoporosis.

In the 1970s, osteoporosis and fractures were not considered a disease, but part of normal aging, and no drug was used for osteoporosis.^27^ Thygesen et al. reported back and muscle pain in some former concentration camp prisoners, probably perceived as symptoms provoked by work, and they considered painful joint an irrelevant symptom.^10^ Considering that bone decalcification can cause back trouble, and that muscle pain can be a sign of osteoporosis, it is probable that the findings of Thygesen et al. indicate muscle and back pain as physical effects of osteoporosis. In line with this study, back trouble (68%) and evidence of osteoporosis in Norwegian concentration camp survivors were found by Lonnum in an earlier study.^28^

Weisz assessed the osteoporosis risk in Holocaust survivors, their children, and grandchildren.^15^ Despite the small sample size (*n*=26), the author concluded that there is a higher risk of osteoporosis in Holocaust survivors. Despite adequate nutrition, the osteoporosis risk was also increased in their first- and second-generation descendants. The author suggested that nutritional deprivation can lead to epigenetic changes and inheritable predisposition to osteoporosis.^15^,^29^–^31^ Supporting this concept, the generational transfer of metabolic insult and onset of osteoporosis has also been reported.^2^ Additionally, other data have shown the direct association between starvation during pregnancy and the degree of bone demineralization in later life.^18^

Another study carried out in a limited number of Holocaust survivors in Australia suggests an increased risk of osteoporosis in survivors exposed to starvation in either childhood or early adulthood.^21^ In addition, the risk of falling and having a hip fracture is increased in Holocaust survivors.^16^,^18^,^23^ Furthermore, the lower discharge FIM scores in Holocaust survivors adversely affect their rehabilitation following hip fracture.^23^ In a self-reported study in Israel that included 530 Holocaust survivors, an increased prevalence of hip fractures versus controls was demonstrated (11. 4% versus 3.9%, respectively).^19^

All these studies underline the importance of either nutrition or environmental conditions on bone health during early life. Despite the limited number of studies in the literature and the limited cohort sizes, all the studies indicate an increased risk of osteoporosis in later life following starvation in early life. In addition, survivors suffered long-term depression during and after the Holocaust. Different studies have reported an increased risk of osteoporosis and hip fracture in depression.^32^,^33^

In line with these findings, Weisz reported the increased risk of osteoporosis in the Israeli population, and the need to raise awareness in the next three decades to prevent osteoporosis, especially in female Jewish immigrants.^34^ All Holocaust survivors and their descendants worldwide could equally be at risk. Lucas reported the effect of nutritional deprivation in pregnancy and in the postnatal period in “programming” future bone demineralization and osteopenia.^35^ Weisz and Albury showed that the pre- and postnatal nutritional deprivation has a “programming” effect on either glucose or lipid metabolism, which is also detected in osteoporosis development.^22^ Early diagnosis and treatment are fundamental for preventing osteoporosis. Of great importance, immediate overfeeding to “compensate” for malnutrition or starvation in early life could lead to metabolic syndrome, including osteoporosis.^33^ These findings suggest the specific need for nutrition education programs for Jewish immigrants. In addition, osteoporosis is a public health concern worldwide for Holocaust survivors and their descendants. Hence programs for preventing osteoporosis should be implemented.

## CONCLUSION

Despite the limited number of papers assessing osteoporosis in Holocaust survivors and their descendants, all of them showed an increased risk in this population. Different bone parameters were assessed in the respective studies.

Of note, only a few studies carried out in a very limited number of patients assessed the transgenerational inheritance of osteoporosis in Holocaust survivors’ descendants. Considering that the etiology of osteoporosis is multifactorial, a larger cohort of offspring and further data on descendants’ risk of osteoporosis are needed.

The results of this study should raise awareness on the potential increased risk of osteoporosis in Holocaust survivors, and especially in the second- generation survivors. Regular BMD screening should be carried out in this population group. The early diagnosis would improve their quality of life, provide palliative care, and ensure proper supplementation with vitamin D, calcium, and pharmacological treatment (teriparatide, bisphosphonates, denosumab, etc.).

Information should be provided to primary care health workers in different countries on the potential increased risk of osteoporosis in Holocaust survivors who have experienced severe nutritional deprivation, and their descendants. Since first-generation Holocaust survivors are increasingly fewer, the primary and current concern is the risk of osteoporosis in the second generation. Potentially, this group of patients can place a significant burden on healthcare systems if not identified and treated. Screening of Holocaust descendants for vitamin D and calcium deficiency could lead to immediate treatment, or to preventive measures to combat bone demineralization.

## Abbreviations

BMD: bone mineral density
DEXA: dual-energy X-ray absorptiometry
FRAX: fracture risk assessment tool
FIM: Functional Independence Measure™
GARVAN: Garvan Fracture Risk™
MRM: members of the resistance movement
PTSD: post-traumatic stress disorder
